# Casemanagers Positioned as Key Advance Care Planning Conversationalists in Oncology Care: A Qualitative Interview Study on the Perspectives of Healthcare Professionals and Patients

**DOI:** 10.1002/cam4.71195

**Published:** 2025-09-04

**Authors:** Anouk Putker, Marieke Perry, Jelmer Chalkiopoulos, Annika Piek, Yvonne Engels, Evelien Kuip

**Affiliations:** ^1^ Radboudumc Nijmegen the Netherlands

**Keywords:** advance care planning, cancer, casemanagers, nursing, oncology, palliative care, qualitative research, shared decision making

## Abstract

**Introduction:**

The increasing global cancer burden and advances in treatments have extended patients' life expectancy, leading to greater prognostic uncertainty and a higher demand for palliative care. Advance care planning (ACP) is essential in this context, ensuring that patients' future care aligns with their values and preferences. Oncology case managers, being nurses with specialized training, have emerged as key ACP conversationalists. This study explores the perspectives of healthcare professionals and patients on case managers as ACP conversationalists for advanced cancer patients.

**Methods:**

A qualitative interview study was conducted with 12 patients who recently had an ACP conversation with their case managers and 12 healthcare professionals, including medical oncologists and case managers, at a university medical center in the Netherlands. Transcripts of semi‐structured interviews were analyzed using inductive thematic analysis.

**Results:**

Three main themes emerged: (1) Trust as the foundation for effective ACP, where patients valued the casemanagers' familiarity and accessibility; (2) ACP aligns with the competencies and tasks of the casemanager, as their communication skills and disease knowledge were seen as strengths; (3) Challenges related to responsibility around treatment decisions and the reluctance of medical oncologists to relinquish control of the conversation. Both patients and professionals recognized the importance of casemanagers having ACP conversations.

**Conclusions:**

Casemanagers are well‐positioned to lead ACP conversations in oncology care, offering trust, continuity, and expertise. Despite some concerns regarding responsibility and medical decision‐making, their role can improve the quality of ACP, supporting more personalized and timely palliative care.

## 1 Introduction

With the expanding global cancer burden and new life‐prolonging treatments, the demand for palliative care increases [1, 2]. Especially, immunotherapy has revolutionized the field of oncology over the last decade by potentially extending the last phase of life substantially [3], and thereby likely increasing prognostic uncertainty and potential symptom burden [4]. This necessitates prolonged palliative care.

Palliative care is an interdisciplinary caregiving approach, aimed at optimizing the quality of life of patients with life‐limiting illnesses [5]. It involves identification, treatment, and support in case of physical, psychosocial, and spiritual discomfort, including anticipation of possible future scenarios, and is highly personal and tailored to individual needs. An essential part of palliative care is advance care planning (ACP) [6]. ACP is a process that supports patients in understanding and sharing their personal values and life goals and helps ensure that people receive future care that is consistent with their preferences [7, 8]. If executed timely, advance care planning has proven to improve the quality of life, increase the chance of dying at the preferred location, and lower health care costs [9, 10]. In 2017, the European Association of Palliative Care recommended ACP application/implementation in daily clinical practice [11]. Since then, many European countries have initiated ACP implementation, including the Netherlands [12, 13, 14, 15].

In current practice, ACP approaches are regularly ad hoc, and most conversations are held within primary healthcare [16]. However, recent literature suggests that in oncology care, GPs prefer the initiation of ACP within the hospital setting. This is due to the GP's inability to oversee treatments, prognosis, and possible future scenarios in a landscape of rapidly evolving treatment options [17]. Additionally, the patient is often temporarily out of the GP's sight during the treatment process in the hospital. Medical oncologists, however, face multiple challenges in having ACP conversations such as time constraints, fear of diminishing hope, and for some, a lack of palliative care skills or ACP training [18]. Recent research has highlighted that ACP involves multiple stakeholders across different care settings, each with distinct roles and expectations, underscoring the need to align ACP with both patient and professional preferences [19].

Oncology casemanagers could fulfill a central role in ACP with patients with cancer, as they already have a central and continuous role during a patient's course of the disease. In the Dutch healthcare system, they are registered oncology nurses who are trained to navigate patients through their disease trajectories and help them understand their disease and treatment options. While previous nurse‐led studies have primarily focused on the outcomes of ACP conversations—such as documentation, place of death, or alignment with patient preferences—little attention has been paid to the interpersonal and contextual factors that shape these conversations [20, 21]. Therefore, this qualitative interview study explored the perspectives and experiences of healthcare professionals and patients on casemanagers positioned as key ACP conversationalists for patients with advanced cancer. By focusing on the unique role of oncology casemanagers, this study aims to contribute to a more nuanced understanding of how ACP can be effectively integrated into practice.

## 2 Methods

### 2.1 Study Design

A qualitative interview study using semi‐structured interviews was performed by interviewing patients with advanced cancer as well as their healthcare professionals on the topic of casemanagers as key conversationalists in advance care planning. The COREQ criteria (COnsolidated criteria for REporting Qualitative research) were followed in reporting the results [22]. Three participant groups were included—patients, casemanagers, and medical oncologists—to obtain a comprehensive understanding of the dynamics and perceptions surrounding ACP conversations. Since ACP is a relational and interdisciplinary process, including all key actors allowed for data triangulation and a fuller picture of how casemanagers are experienced and positioned in practice.

The research group had different backgrounds and knowledge, which facilitated a broad view due to different perspectives on the thematic analysis. The research group consisted of a senior researcher/medical oncologist/palliative care specialist (EK, female), a professor in meaningful healthcare (YE, female), a senior researcher/general practitioner (MP, female), a PhD candidate/medical oncologist in training (AEP, female) and two medical students (AP, female and JC, male). The two medical students (AP and JC) had basic knowledge of qualitative research but were strictly supervised by experienced qualitative researchers. Their contributions were continuously reviewed in team meetings to ensure methodological rigor. The senior researchers are trained and experienced in qualitative research methodology.

### 2.2 Setting

This study was performed within the outpatient clinic of the department of Medical Oncology of the Radboud university medical center; an academic hospital in the southeastern part of the Netherlands. In this hospital, oncology casemanagers (hereinafter referred to as ‘casemanagers’) are assigned to all patients with cancer. Casemanagers are easily accessible, have an informal and personal patient approach, and have extensive disease‐ and treatment‐specific knowledge. Casemanagers are not authorized to make treatment decisions. Their post‐graduate education also includes the principles of palliative care and ACP based on national guidelines. Casemanagers are integral members of the oncology care team and can form the bridge between patients and their healthcare professionals [20]. Recently, casemanagers in the department started having ACP conversations with patients. The content was based on the Dutch ACP guideline and included topics on the existential, psychosocial, and physical domains [23]. The structure and topics that were discussed during the ACP conversations are provided in Appendix S1 [23].

## 3 Study Population

Convenience sampling was applied to recruit patients. Eligible patients were identified by their casemanager based on recent participation in an ACP conversation and were approached directly by the casemanager or a member of the research team. To ensure accurate recollection and minimize recall bias, only patients who had participated in an ACP conversation within the previous 4 weeks were included.

The reason for the ACP conversation was either disease progression, severe deterioration in quality of life, or at the patient's request. Next, the healthcare professionals that were associated with the included patients were approached. No medical records were reviewed for recruitment.

Each interviewee was provided with an information letter and informed consent form by a member of the research team. Interviewees were given several days to review and contemplate the information presented in the forms. Participation was entirely voluntary, with no consequences for declining or withdrawing. No coercive methods were used, and the confidentiality of the participants was strictly maintained.

### 3.1 Participant Characteristics

A total of 24 participants were approached and all consented to participate in the interviews. The inclusion and exclusion criteria for participant selection are detailed in Table 1. The healthcare professional group consisted of 12 individuals with a mean age of 43 years (range: 33–56), of whom 92% were female (*n* = 11). This group included 5 case managers and 7 oncologists (of whom some in training), all possessing several years of experience in the oncology field.

**TABLE 1:** Inclusion and exclusion criteria for participants.

Inclusion criteria patients (*n* = 12)	Inclusion criteria healthcare professionals (*n* = 12)	
	Casemanager (*n* = 5)	Medical oncologist (in training) (*n* = 7)
Advanced (incurable and/or metastatic) cancer ACP conversation with casemanager < 4 weeks ago	≥ 1 ACP conversation with a patient < 4 weeks ago Written and oral information transfer to the medical oncologist (in training)	Physician received ≥ 1 written and oral information transfer from the casemanager and discussed its content with the patient
Exclusion criteria: not proficient of Dutch language or not fit enough for an interview		

The patient group also consisted of 12 individuals, with a mean age of 67 years (range: 57–77 years), and 58% were female (*n* = 7).

### 3.2 Data Collection

All participants in this study had previously engaged in an advance care planning (ACP) conversation. Patients were provided a comprehensive explanation of ACP prior to the conversation by the case manager. Consequently, a detailed introduction to ACP prior to the interviews was not deemed necessary. However, when clarification was required, additional explanations were provided, ensuring that participants understood the concept of ACP within the context of a patient's values, sources of strength, psychosocial circumstances, communication needs, and preferences regarding future care.

Semi‐structured interviews were held with the use of a topic list (Appendices S2 and S3). The topics were based on discussions within the research team and themes extracted from literature [24, 25]. The topic list included introductory questions about participants' general views on ACP to help them reflect and ease into the conversation. These questions served as a starting point to explore their experiences with case managers as ACP conversationalists.

Patients were interviewed by researcher AP and healthcare professionals by researcher JC. The interviews were face‐to‐face and held in Dutch. The duration of the interviews varied between 30 and 55 min and no repeated interviews were held. The first two interviews were practiced and discussed with the research team. All interviews were audiotaped, transcribed verbatim, and pseudonymised. Data saturation was frequently discussed in meetings within the research team. Enrollment of participants ended when data saturation was reached and no new concepts emerged.

#### 3.2.1 Data Analysis

Inductive thematic analysis was applied to the transcripts. ATLAS.ti (version 24.0.0) was used to support this process. Analysis started after all interviews were held. Two researchers (AP and JC) independently coded the first six interviews as closely to the transcript text as possible to minimize subjectivity. Codes were compared and discussed within the research team until consensus was reached on two codebooks (patient interviews and healthcare professionals interviews). For each codebook, open codes were combined into axial codes and themes. Based on comments from the research team, researcher AEP re‐read all interviews, codes, and themes and reformulated categories and themes and translated them to English. The analytic process was thoroughly discussed in research meetings where drafts were refined or provided with new input as needed.

### 3.3 Credibility of the Data

The interviewers (AP and JC) did not have any relationship with the participants prior to the study, which lowered the chance of socially desirable answers. Participants were given the rationale for the study (so they could provide informed consent), but they did not know the interviewers' goals. Lastly, only the interviewer and the participant were present during the interviews.

Investigator triangulation was applied by involving multiple researchers in data interpretation, ensuring a more balanced analysis. Data triangulation was applied by involving various professional perspectives on the topic and thereby increasing the robustness of the data.

The participants were given the option to receive interview transcripts and a summary of the findings, but none expressed interest. Transcripts were carefully created and checked with the audio files of the interviews. Patients did not provide feedback on the study findings.

### 3.4 Ethical Approval

The study was performed according to Dutch law and Good Clinical Practice guidelines. The Medical Review Ethics Committee region Arnhem‐Nijmegen concluded this study was not subject to the Medical Research Involving Human Subjects Act (case number CMO 2023‐16609).

## 4 Results

The importance of ACP was universally acknowledged by all participants. The healthcare professionals in this study emphasized that ACP is a process rooted in teamwork. They emphasized that discussions about values, goals, and preferences should not be limited to nurses or physicians, but are rather a collaborative process. Many professionals noted that while some colleagues may naturally excel at these conversations, others might feel out of their element. Therefore, the healthcare professionals suggested that skills might take precedence over organizational hierarchy. Both case managers and medical oncologists (hereinafter referred to as ‘oncologists’) stressed the importance of effective communication and approachable contact between professionals, and consequently, both of them could be the key conversationalists. This was supported by three themes that were identified within the data. In all themes, both patients' and healthcare professionals' perspectives and experiences were represented.

### 4.1 Theme 1: Trust Is the Foundation for Effective ACP

Patients were positive about the casemanager being an ACP conversationalist due to the fact that they are a familiar and trustworthy caregiver. The majority of patients stated that trust is based on the fact that the casemanagers are present during many of the patients' consultations with the oncologists and often afterwards. Patient 08: “It means a lot that she [casemanager] is always there. It is like seeing a familiar face in the crowd.” Some patients stated that trust was established because their oncologist trusted and actively involved the casemanager. Patients felt that the oncologist and casemanager are a team and have different/complementary tasks. Patient 04: “In the consultation room, the doctor sometimes falls silent, and then the casemanager adds to the conversation. You can see that they are truly a close‐knit team.”

A few patients mentioned both the oncologist and the casemanager could be excellent conversationalists and they did not prefer one over the other. Most patients expressed a more personal connection with the casemanager than with the oncologist. The healthcare professionals also emphasized the importance of trust in ACP conversations. Oncologists noted that the casemanagers' continuous presence and informal relationship with patients fostered a level of openness that was often harder to achieve in physician‐led conversations. Oncologist 05: “The casemanager's relationship with the patient is different to ours”.

Several patients mentioned that trust was established because the casemanagers are often easily available for questions, concerns or personal small talk. Patient 11: “She [casemanager] is my point of contact. I do not mind asking my oncologist questions, but I have to wait a lot longer for an answer. When you are concerned, a rapid answer is very important and yes, that establishes trust”. Patient 02 explained: “As a patient you feel really unsafe, you are completely out of control. It is really supporting to know you can contact someone directly, especially in a large institute”.

Patients felt at ease while talking to the casemanager, due to trust that had been built over the months or years. Some patients explicitly mentioned that trust in the casemanager increased their willingness towards ACP. When patients were asked for their needs in ACP many answered with ‘an open and honest conversation’. Patients appreciated the straightforward approach to difficult topics, but pointed out that this approach would not have worked with someone unfamiliar.

All patients reflected on the impact of the ACP conversation. A majority of patients felt empowered and seen by the casemanager. They experienced a growing sense of companionship with the casemanager. Many patients also felt a sense of security regarding future care. Patient 09: “When I look at the three letters ‘ACP’ I think: they will take good care of me”.

### 4.2 Theme 2: ACP Aligns With the Competencies and Tasks of the Casemanager

The casemanagers and oncologists unanimously stated that the casemanagers are well‐equipped for having ACP conversations. The nursing background and their interpersonal and communication skills make them excellent ACP conversationalists. Oncologist 02: “During an ACP conversation, concerns might be addressed regarding possible symptoms, treatment outcomes and prognosis. Without the disease‐specific knowledge these concerns could not be explored sufficiently and future scenarios could not be discussed”. All healthcare professionals expressed that these topics are crucial for providing high quality anticipatory care.

Patients often referred to casemanagers as ‘experts in the field of ACP’. Many patients mentioned the empathetic, understanding and humane approach of the casemanagers during the conversation. Some patients described the casemanager as very skilled at making difficult topics seem ordinary.

Even patients that had an ACP conversation with a less experienced casemanager were mainly positive. Patient 04: “She [casemanager] was struggling, I could see that. Certain topics were difficult for her. If was helpful to see because we are all human, not robots”.

Both the oncologists and casemanagers agreed that having an ACP conversation is not significantly different from the casemanagers' usual tasks. Many ACP topics are already being discussed in daily practice. Casemanager 03 explained: “These conversations sometimes occur casually, such as in the hallway or in a treatment chair. But as a result, they tend to be superficial and are rarely documented properly.” Oncologist 01 adds: “In my opinion, these aren't new topics. The casemanager discussed these topics before, but by labelling it as an ACP conversation and explicitly presenting it as such to the patient, it provides structure and gives the conversation added value.”

Another reason to assign ACP conversations to the casemanagers is that their work allows for a more flexible schedule. All interviewees—including patients—mentioned that oncologists do not have enough time to do everything by themselves. Oncologist 03 confirmed: “I recognize the importance of ACP, but due to the tight clinical schedule and high workload, I lack the time to truly focus on it. The topic hardly gets addressed, which is a shame.” Patients noted that having a dedicated moment to discuss ACP topics was essential. They greatly appreciated the fact that ample time was allocated for the conversation. Patient02: “She [casemanager] took an hour of her time. That felt special to me. The usual conversations in a consultation room with the oncologist last no longer than 10 minutes. Once you close the door after that, you feel completely overwhelmed”.

### 4.3 Theme 3: Challenges Related to Responsibility and Treatment Decisions

Despite the similarity to their usual tasks, casemanagers expressed a sense of uncertainty when having ACP conversations. This did not necessarily refer to the actual content of the conversation, but rather to having a new form of independence and feeling responsible for correctly having and documenting an important conversation. Casemanager 03 explains: “I still feel uncertain in ACP conversations. I must admit that I sometimes wonder if I have the right skills to handle them, especially given how sensitive the topic is.”

Similarly, oncologists voiced concerns regarding the responsibility and potential outcomes of the conversation. Oncologist 05 reported “I don't feel I can let go of things yet”. The oncologists seemed particularly worried that casemanagers might not possess the same medical expertise as physicians, which could impact the quality of the conversations. Oncologist 03: “Topics outside of their expertise might be discussed”. A number of oncologists expressed difficulty in handing over the conversation, as they were concerned that topics such as current cancer treatments, end‐of‐life care and possible emergency life support might arise. Oncologist 03: “These topics require caution, nuance and expertise”. Although these topics could naturally arise in the conversation, many patients mentioned they had no intention to make treatment decisions in the ACP conversation with the casemanager. Patient 08 talked about treatment boundaries: “I know that she [casemanager] is not in charge of that. I don't want that either. It actually feels liberating to be able to talk openly and brainstorm without being afraid that something will be attached to it.” All interviewees agreed that medical decision‐making belongs to the oncologist together with the patient. Numerous patients expressed that possible treatment decisions hamper an open ACP conversation with the oncologist. Patient 06: “It is hard to be completely honest with the oncologist because I fear he [oncologist] will stop or change my current treatment.” Analogously, oncologists agreed that not making treatment decisions makes casemanagers more approachable. Oncologist 06 illustrates: “The question, which was an open one to explore his [patient] situation, was immediately linked to potential treatment decisions on my part. I believe that with a casemanager, the patient feels much closer to themselves and is not focused on what happens with the answers on the other side of the table.”

Meanwhile, the majority of the oncologists agreed they themselves do not talk about treatment boundaries often enough. Oncologist 04: “Usually there is no immediate reason to talk about treatment preferences and boundaries”. Oncologists also struggled with documenting possible boundaries because they are not black‐and‐white. Oncologist 10: “What is acceptable to a patient can vary greatly in the last phase of life. It might change too”. The majority of oncologists agreed that the ACP conversations conducted by the casemanagers provided more context around the patient than their own conversations and therefore gave opportunity to talk about treatment preferences earlier on.

## 5 Discussion

### 5.1 Main Findings

This study demonstrates that implementing ACP conversations performed by oncology casemanagers is greatly valued by patients with advanced cancer, oncologists and the casemanagers themselves, highlighting the collaborative nature of these interactions among healthcare professionals and patients. This interview study revealed that trust is a critical component for successful ACP, a finding that is consistent with existing literature [26, 27, 28, 29]. Despite this finding, several hospitals in the Netherlands have established ‘ACP outpatient clinics’, where patients meet with a specialized nurse or physician, with whom they have no professional relationship, to address ACP‐related topics. In superlative, several studies were recently performed using web‐based programmes as ACP tool without human interaction [30, 31]. These studies had high drop‐out and refusal rates, possibly suggesting the importance of personal contact and trust. Our findings strongly advocate for incorporating ACP conversations within the context of a patient's established treatment team, as this is likely to foster a more open and honest dialog. Patients expressed a strong preference for casemanagers as key ACP conversationalists due to their established relationships as familiar and trustworthy caregivers. Recent literature also shows that patients often prefer ACP conversations to be initiated by non‐physician professionals, and that these conversations are more effective when embedded in routine care rather than triggered by acute changes in health status. This supports the positioning of casemanagers as trusted and accessible ACP conversationalists [19].

Additionally, our study identified how ACP conversations align with casemanagers' usual tasks. Both patients and healthcare professionals acknowledged the competencies of casemanagers in facilitating ACP. Their nursing backgrounds and strong interpersonal skills enable them to effectively address complex issues [32, 33], with patients often referring to them as “experts” in ACP. Despite recognizing the similarities between ACP conversations and their routine tasks, our study revealed challenges related to responsibilities and treatment decisions when casemanagers performed ACP conversations. Casemanagers expressed uncertainty about their newfound responsibilities in these conversations, a result that is in line with existing literature [6, 34]. This sense of responsibility likely contributed to ensuring that these important conversations were correctly conducted and documented. Similarly, oncologists shared concerns regarding the implications of casemanagers having ACP conversations, fearing that critical treatment decisions might be intertwined within the conversations. This phenomenon is consistent with existing literature where ACP is used to support or accelerate decision‐making [35, 36]. However, the use of casemanagers as ACP conversationalists more closely aligns with the original intention of the concept: identifying wishes, values, and needs [37]. Both oncologists, casemanagers, and patients concurred that while treatment preferences can be discussed with a casemanager, these conversations should remain exploratory in nature.

This study highlights an interesting paradox in ACP. On the one hand, ACP topics are rarely addressed in everyday clinical practice [38, 39, 40, 41], yet oncologists simultaneously express concerns about delegating responsibility for these critical conversations to case managers. The main barrier cited for not engaging in ACP conversations appears to be time constraints, a result that echoes previous findings [18, 42, 43]. This paradox points to a pressing need for a cultural shift, where physicians must embrace the delegation of tasks to enhance the overall quality of care. This necessary shift, however, has not yet been explored in the existing literature.

Overall, these findings underscore the importance of trust, communication skills, and role clarity in ACP conversations, positioning casemanagers as essential conversationalists who empower patients while navigating the complexities of advance care planning. The interplay between patient and casemanager is unique and impossible to match by a doctor due to the different roles they occupy.

### 5.2 Strengths and Limitations

This study offers valuable insights into the perspectives of healthcare professionals and patients regarding the role of case managers in ACP. One of its strengths is the inclusion of diverse viewpoints (data triangulation), capturing a comprehensive understanding of the ACP process. The qualitative methodology, utilizing semi‐structured interviews, allows for in‐depth exploration of participants' experiences, resulting in rich and nuanced data. Investigator triangulation was applied by involving multiple researchers in data interpretation, promoting a more balanced analysis and trustworthiness of study results.

Conducted within a specific healthcare setting, this study reflects the realities of ACP conversations in the outpatient oncology context, making the findings relevant for healthcare professionals operating in similar environments. The most important limitations are related to the transferability of the study results, including its selection from an academic hospital. The female population was overrepresented in the group of healthcare professionals, but given the growing gender imbalance in healthcare, this reflects reality [44].

### 5.3 Future Implications for Clinical Practice and Research

This study showed that using the expertise and skills of the case manager in ACP creates in‐depth context, preferences, and values around a patient. This generates an opportunity for the oncologist to revisit important findings, which give room for possible discussions about treatment preferences and boundaries. Within this setting, oncologists had a smaller, yet equally important role in ACP. We found a strong willingness to incorporate this joint effort in daily practice, but oncologists remain somewhat hesitant due to their concerns on responsibilities, especially regarding medical decision‐making. This study emphasizes the need for proper national or local guidelines on role clarification and responsibilities.

As the need for ACP grows, there is also an increasing demand for ACP conversationalists. Meanwhile, significant shortages in hospital staff and general practitioners are predicted [45]. Positioning the casemanager as the key ACP conversationalist could improve the quality of care and enhance efficiency. Several studies have shown the potential reduction of inappropriate end‐of‐life care by using ACP [21, 46, 47]. This suggests that investing in ACP could save on time and the deployment of staff later on. Future studies are needed to evaluate time and cost effectiveness as well as long‐term effects of ACP by casemanagers to enhance further implementation.

## 6 Conclusion

This study highlights the pivotal role of case managers as key conversationalists in ACP for patients with advanced cancer. Healthcare professionals and patients recognized the importance of trust, effective communication, and collaboration in ACP. Case managers, already knowing the patients well and with their nursing competencies and interpersonal skills, are uniquely positioned to facilitate these conversations. With a clear division of roles and responsibilities regarding medical decision‐making, case managers should be integrated into the ACP process. The use of case managers strikes a balance by exploring a patient's wishes, values, and needs while preserving the oncologist's responsibility in decision‐making. This joint effort contributes to a more comprehensive and patient‐centered approach to advance care planning.

## Author Contributions

**Anouk Putker:** formal analysis (lead), investigation (lead), project administration (lead), software (lead), writing – original draft (lead), writing – review and editing (lead). **Marieke Perry:** data curation (equal), formal analysis (equal), supervision (equal), validation (equal), writing – review and editing (equal). **Jelmer Chalkiopoulos:** formal analysis (supporting), formal analysis (supporting), investigation (supporting), investigation (supporting). **Annika Piek:** formal analysis (supporting), investigation (supporting). **Yvonne Engels:** conceptualization (equal), data curation (equal), methodology (equal), resources (equal), supervision (equal), validation (equal), visualization (equal). **Evelien Kuip:** conceptualization (equal), data curation (equal), formal analysis (equal), funding acquisition (equal), investigation (equal), methodology (equal), project administration (equal), supervision (equal), validation (equal), visualization (equal), writing – original draft (equal), writing – review and editing (supporting).

## Conflicts of Interest

The authors declare no conflicts of interest.

## Supporting information


**Appendix S1:** cam471195‐sup‐0001‐Appendix1.docx.

**Appendix S2:** cam471195‐sup‐0002‐Appendix2.docx.

**Appendix S3:** cam471195‐sup‐0003‐Appendix3.docx.

## Data Availability

The data that support the findings of this study are available upon reasonable request from the corresponding author.
