# Supplementary material for: Casemanagers Positioned as Key Advance Care Planning Conversationalists in Oncology Care: A Qualitative Interview Study on the Perspectives of Healthcare Professionals and Patients
Source: Cancer Med. 2025 Sep 4;14(17):e71195. doi: 10.1002/cam4.71195 (PMC12409654; doi:10.1002/cam4.71195)
Supplement: Supplementary file 1 — Appendix S1: cam471195‐sup‐0001‐Appendix1.docx. [file CAM4-14-e71195-s002.docx]

**Appendix 1:**Content of the ACP conversation

| Patient context | “For the patient it is essential to…” |
| --- | --- |
|  | “Are there any specific events or goals that the patient would like to achieve or experience?” |
|  | “Which individuals are significant to the patient?” |
|  | “What is a source of strength for the patient? Does it still succeed in this situation? Are there additional needs in this regard?” |
|  | “Are there issues regarding the patient’s context that could pose a problem now or potentially in the future? Consider roles, tasks or financial aspects.” |
| Psychological functioning | “Are there particularities concerning psychological functioning, such as mental health issues, delirium, dementia or questions regarding capacity for decision-making?” |
| Communication | “What is the proficiency level in understanding Dutch?” |
|  | “Is assistance needed in communication? For example, considering hearing impairment.” |
|  | “What is the level of internet proficiency?” |
| Healthcare organization | “Are there informal caregivers? Consider children, neighbors, etc.” |
|  | “How is the communication with the GP?” |
|  | “Involved transmural disciplines (consider home care, physiotherapist etc.)” |
|  | “What is currently required for the patient to continue functioning optimally? Are there concerns about vitality or mobility that the patient is currently facing?” |
|  | “Assistive devices: …” |
| Treatment wishes and limitations | “Are the goals of the current care clear for the patient? Should these be reconsidered in discussion with the healthcare provider if necessary?” |
|  | “Experiences with illness, loss or fear; specific preferences or aversions for the patient? Are there particular concerns or fears, present or in the future?” |
|  | “Wishes regarding treatment preferences or limitations: preferences for the location of care, place of death? Have these wishes been documented anywhere or is this still needed?” |
| Evaluation ACP conversation | “Key points from the ACP conversations:” |
|  | “Follow-up discussion with the oncologist” |
