# Supplementary material for: Casemanagers Positioned as Key Advance Care Planning Conversationalists in Oncology Care: A Qualitative Interview Study on the Perspectives of Healthcare Professionals and Patients
Source: Cancer Med. 2025 Sep 4;14(17):e71195. doi: 10.1002/cam4.71195 (PMC12409654; doi:10.1002/cam4.71195)
Supplement: Supplementary file 2 — Appendix S2: cam471195‐sup‐0002‐Appendix2.docx. [file CAM4-14-e71195-s001.docx]

**Appendix 2:
Interview topics list for patients**

The main questions of the interview are:

• “How did you first feel about going into an ACP conversation?”
• "What were your experiences with advance care planning conversations with your case manager?”
• "What approach did your casemanager take when discussing ACP with you?"
• "Looking back at your experience with ACP conversations, what are your overall thoughts and feelings about the process? What aspects did you appreciate, and which did you find challenging?"

To ensure the interview has a clear and logical structure, the main and sub-questions will be organized into 3 distinct phases:

**1. Introduction to ACP:**

a. What motivated the patient or caregivers to initiate an ACP conversation? Who took the first step?
b. How did you feel when the need for this conversation was introduced?
c. How did you feel about the preparation for this conversation? Did you feel adequately prepared?
d. What were your thoughts and feelings about the ACP conversation before it took place? What expectations did you have?

**2. Experience of the ACP Conversation:**

a. Can you describe what your ACP conversation was like?
b. What has been your overall experience with advance care planning discussions with your casemanager? Did you encounter any challenges or barriers during these conversations? What aspects of the conversation were helpful?
c. How did you feel about your case manager’s approach during the conversation?
d. Do you think the casemanager was the right person to have this conversation with you?

**3. After the Conversation:**

a. How did the ACP conversation impact your understanding of your disease trajectory?
b. Did you discuss the outcome of the ACP conversation with your medical oncologist? If so, how did that discussion go?
c. Did you share the outcome of the ACP conversation with your general practitioner? If so, how did that conversation unfold?
d. Looking back, what are your overall thoughts and feelings about the ACP process and its impact on you?
e. How did the conversation compare to your expectations?
f. Would you recommend that other patients have a similar conversation?
