# Supplementary material for: Casemanagers Positioned as Key Advance Care Planning Conversationalists in Oncology Care: A Qualitative Interview Study on the Perspectives of Healthcare Professionals and Patients
Source: Cancer Med. 2025 Sep 4;14(17):e71195. doi: 10.1002/cam4.71195 (PMC12409654; doi:10.1002/cam4.71195)
Supplement: Supplementary file 3 — Appendix S3: cam471195‐sup‐0003‐Appendix3.docx. [file CAM4-14-e71195-s003.docx]

**Appendix 3:
Interview topics list for casemanagers**

The main questions of the interview are:

- How does the casemanager feel before the conversation? (For example, anxious, well-prepared, or stressed.)
- To what extent does the casemanager feel equipped and comfortable to lead these ACP conversations?
- What outcomes have these conversations produced?

To ensure the interview has a clear and logical structure, the main and sub-questions will be organized into 2 distinct phases:

**Experience with the conversation:**a. How do you feel about conducting these conversations?
b. How much experience did you have with conducting ACP conversations before this research began?
c. How did you prepare for these conversations (e.g., training, information, literature)?
d. How did the conversation go? (This could refer to a specific conversation or be more general.) Did it achieve the desired outcome? What was your impression of how the patient and their loved ones felt about it?
e. In your view, what makes a conversation successful?
f. Who do you think is the most suitable person to lead these conversations?
g. How did conducting these conversations affect you personally?
h. Did you face any challenges during the conversations? If so, what were they?

**Experience with the care process:**a. How did the conversation contribute to the overall care process? Do you consider it beneficial?
b. How is it to provide feedback about the conversation to the treatment team?
c. How did you report the outcomes of these conversations?
d. Were you able to gather relevant information about what is meaningful for the patient and integrate it into their care preferences?
e. Are there aspects you would like to see improved (e.g., more training, specific types of training, more/less time, number of conversations, or the format followed)?

**Interview topics list for medical oncologists:**

The main questions of the interview are:

- To what extent does the oncologist (in training) believe the casemanager is adequately equipped to conduct these ACP conversations?
- What have the conversations yielded?
- How does the oncologist (in training) perceive the way casemanagers conduct these conversations?
- How is the information made accessible after the conversations? Is it clear and easy to understand?

To ensure the interview has a clear and logical structure, the main and sub-questions will be organized into 2 distinct phases:

**1. Experience with handing over the conversations:**
a. How do you feel about the casemanager taking a larger role in these conversations?
b. Do you believe the casemanager is the most suitable person to conduct these conversations?
c. When do you consider a conversation to have been valuable?
d. Did you face any challenges because the casemanager led the conversation? If so, what were they?

**2. Experience regarding the care process:**
a. How did you receive feedback from the conversation (without having conducted it yourself)?
b. How were you able to review the information gathered from the conversations afterward?
c. Were you able to extract relevant context (information about what is meaningful to the patient) from the reports and integrate it into their preferences?
d. How did the conversation contribute to the care process? Do you consider it beneficial?
e. Are there aspects you would like to see changed? (For example, more training—if so, what kind? More or less time allocated? More or fewer conversations? Changes in format? Or perhaps conducting the conversations yourself?)
